# Applicability of the Child Health Utility instrument to measure health utility in children with intestinal failure: A qualitative study of caregivers

**DOI:** 10.1016/j.intf.2024.100024

**Published:** 2024-11-05

**Authors:** Vikram K. Raghu, Lisa Lakkis, Flor de Abril Cameron, Daniela Gattini Valdes, Beverly Kosmach-Park, Janel Hanmer

**Affiliations:** a University of Pittsburgh School of Medicine, Pittsburgh, PA, USA; b UPMC Children’s Hospital of Pittsburgh, Pittsburgh, PA, USA; c Institute of Health Policy, Management and Evaluation, University of Toronto, Toronto, Ontario, Canada; d Hospital for Sick Children, University of Toronto, Toronto, Ontario, Canada

**Keywords:** Quality of life, Intestinal rehabilitation, Short bowel syndrome, Pediatrics

## Abstract

**Background:**

Caregivers of children with intestinal failure make many difficult decisions without quantitative data to support how those decisions affect quality of life (QoL). To determine if the Child Health Utility instrument (CHU9D) could be used to collect standardized QoL data, the study aims to determine the most important QoL domains for caregivers of children with intestinal failure.

**Method:**

Semi-structured interviews were completed with caregivers of children with intestinal failure that focused on their perspectives of QoL. A content analysis was performed to determine if caregiver perspectives aligned with the nine domains of the CHU9D to support its use in children with intestinal failure.

**Results:**

There were 10 participants in the study: 9 biological mothers and 1 biological father. Indications for intestinal failure included short bowel syndrome (n = 8), congenital enteropathy (n = 1), and intestinal dysmotility (n = 1). Caregivers endorsed all 9 domains as important with 77.8 % of domains having unanimous agreement. Annoyance and tiredness were each recognized as unnecessary by two participants. Without prompting, most caregivers described QoL through the domains of sadness, daily routine, and activities. When a subgroup of 8 participants were asked specifically about which domains were most important, sadness was cited by the majority (7/8) of caregivers.

**Conclusion:**

The caregiver perspective of important domains for assessing QoL is well-reflected through use of the CHU9D. Opportunities exist for examining whether fewer domains may be sufficient, Future work should confirm these findings directly with children with intestinal failure.

## Highlights

• Parents of children with intestinal failure report domains of quality of life that are represented in the CHU9D.
• Sadness alone may be the most important quality-of-life domain to consider.
• Activities, daily routine, and school can serve as excellent surrogates for assessing quality of life.

## Introduction

Children with intestinal failure are faced with many challenging decisions with little guidance. As the survival for these children without the need for transplant has improved over time, the focus has shifted towards decision making that may improve quality of life (QoL). Caregivers cite QoL as an important factor in decision-making, yet quality of life measures in pediatric intestinal failure lack data on validity and reliability [1], [2]. Furthermore, there is a lack of validated preference-based health-related QoL or health utility measurements, which are required for the calculation of Quality-Adjusted Life Years (QALYs) [3], [4], [5], [6], [7].

Health utility measurement produces quality-of-life weights between 0 and 1, where a weight of 1 represents perfect health while 0 represents a state equivalent to death [8]. The strength of health utilities compared to other forms of quality-of-life measurement lies in the ability to use these weights across various conditions and to evaluate the economic impact of disease. Currently, the only existing health utility study in intestinal failure correlated health utility to days of parenteral nutrition use, but the results are inconsistent with the way pediatric patients describe quality of life [9]. For example, the study suggested the quality of life with 7 days of parenteral nutrition per week was less than that of paraplegia [10].

Several instruments exist to measure health utility in children, but none have been used or validated in pediatric intestinal failure. The Child Health Utility instrument (CHU9D) was developed specifically for the pediatric population, with nine QoL dimensions that are relevant for this age group [11], [12]. This instrument is concise, easy to administer, and has excellent psychometric properties in children ranging from 7–17 years with a wide range of acute and chronic health conditions [11], [12], [13], [14]. However, the CHU9D does not have evidence of reliability, validity, and responsiveness in children with intestinal failure, a complex patient population that has unique medical needs. While validity has many components, content validity, defined by whether an instrument covers all relevant domains of a concept, plays a particularly important role for quality of life [15]. If the tool is to be used for decision-making in the intestinal failure population, then the domains should reflect the priorities of these families.

The aim of this study was to assess the content validity of the CHU9D to measure QoL in pediatric intestinal failure.

## Materials and methods

We performed a qualitative analysis of semi-structured interviews of caregivers of children with intestinal failure to determine which domains of QoL were most important to them and whether the nine domains of the CHU9D were appropriate for children with intestinal failure. Qualitative research focuses on systematically obtaining the viewpoints of a specific individual of group by collecting information often via free response. These data may then be analyzed for explicit content or implicit themes that arise. The power of qualitative analysis lies in the ability to express similar viewpoints among participants while simultaneously highlighting divergent comments. Qualitative methods have previously been used to determine content validity for other instruments [16]. This study was approved by the local Institutional Review Board (STUDY21100136). Verbal consent was obtained from all participants.

Caregivers were recruited from the local intestinal rehabilitation clinic if they had a child followed at the clinic receiving parenteral nutrition and could participate in an English-language interview. Each caregiver participated in a half-hour semi-structured interview via Zoom with an interviewer trained in performing qualitative interviews. Interviews focused on topics such as what QoL means, what determined QoL in their children, and the interaction between their own and their child’s QoL. After an open-ended question about determinants of QoL, caregivers were prompted with each of the nine domains in the CHU9D and asked whether these were factors the healthcare team should think about when considering QoL. The domains included worry, sadness, pain, tiredness, annoyance, school, sleep, daily routine, and activities. Interviews were auto-transcribed by Zoom and then edited for accuracy using the audio recording by the research team. Interviews were conducted until thematic saturation was achieved with a minimum plan for nine interviews based on the minimum necessary to reach thematic saturation [17].

The primary analysis was a deductive analysis to determine whether the nine domains of the CHU9D were felt to be indicators of QoL by the caregivers. Secondary analysis involved a comparison of indicators spontaneously stated by caregivers versus those that required prompting as well as those indicators which were identified as most important by caregivers. Each of the nine domains was assigned a code; no other codes were used. Interviews were coded using NVivo v14 (Lumivero, Denver, CO, USA). Two coders reviewed all transcripts and coded each one. Discrepancies in coding were resolved through unanimous deliberation.

## Results

Twenty-three caregivers were approached to participate in interviews. Three declined. Ten additional caregivers agreed to participate initially but were unable to successfully schedule and complete the Zoom interview. Thus, ten caregivers, all biological parents, participated in the interviews. Nine of the ten were female. Their children predominantly had intestinal failure from short bowel syndrome from various etiologies, but one had microvillus inclusion disease and one had megacystic-microcolon-intestinal hypoperistalsis syndrome. Children were evenly split by parent-stated sex.

**Fig. 1:**
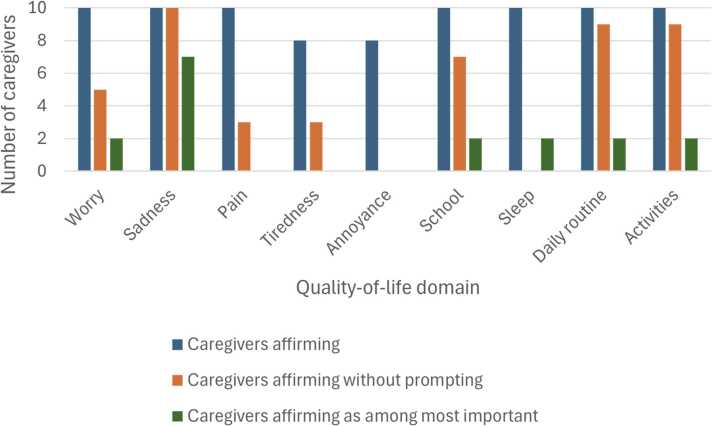
Caregiver-identified priorities in quality-of-life domains for children with intestinal failure.

Fig. 1 shows the number of caregivers that affirmed each of the nine domains as reflective of QoL, the number who mentioned each domain without prompting, and the number who specified each domain as holding higher importance. There were no specific domains mentioned by caregivers outside of the domains listed. When asked directly about the nine domains, all ten caregivers agreed that seven of the nine domains were important to consider when measuring QoL. Regarding how annoyed the child is, two caregivers felt this was unimportant to measure with one stating that “basically every child that's [their child’s] age is gonna be annoyed with everything” (P2). Regarding tiredness, two caregivers neither affirmed nor refuted the importance during their interviews, but both caregivers affirmed that sleep was important. Table 1 shows a representative quote from each of the nine domains.

**Table 1:** Representative quotes from caregivers of children with intestinal failure reflecting the importance of each quality-of-life domain from the Child Health Utility instrument.

Domains	Representative Quote
Worry	“He worries a lot when he gets in the hospital. And he worries about small things here that might cause him to be in the hospital” (P5)
Sadness	“Generally, if a child’s mopey and not happy, I mean, are they really thriving?” (P7)
Pain	“[Good quality of life is] just being able to do those things comfortably, and not, yeah, I guess not experience a lot of pain in the process.” (P3)
Tiredness	“Yeah, cuz if they're sluggish, something's not right. But if they’re bouncing off the walls, they should be okay.” (P2)
Annoyance	“As he's getting older, he's definitely more annoyed and aware that he has to deal with more than most kids his age do, and it's frustrating.” (P10)
School	“She's full participating in a first-grade classroom. And so, the fact that she can go there every day and not miss a lot of school, not be out of school sick, that would be probably one of our, our top priorities.” (P9)
Sleep	“Because I think that if they don’t sleep very well, then they’re cranky and then they don’t want to like work with anything. I just feel like sleep is important.” (P1)
Daily routine	“I definitely think that's important, especially as they get older. You know, being able to take care of yourself, keep, you know, do the things you need to do.” (P8)
Activities	“Just being able to do the things that, you know, people, you know, your own, your age, or, you know, are able to do. Swimming; it was a big thing […] just being able to do age-appropriate things without restrictions.” (P8)

Prior to going through the list of domains in the CHU9D, each caregiver was asked open-ended questions about QoL. In response to open-ended questions, most caregivers cited three domains that they consider, namely happiness/sadness (10/10 caregivers), daily routine (9/10 caregivers), and activities (9/10 caregivers). One participant defined QoL as “just the ability to live a happy […] normal, as normal as possible lifestyle” (P8) while another said that in thinking about a good quality of life, she “think[*s*] about [her child’s] happiness” (P5). One caregiver specified the link between activities and not feeling sad, stating that QoL was about “just [her child’s] happiness, his ability to do normal things, to not feel isolated because of the things that he has that are different; being able to participate in things we do as a family” (P3). Another participant defined QoL as “how well [her child is] able to participate in the daily activities of a six-year-old […] and to what degree her medical diagnosis and care impacts her day-to-day activities and her day-to-day life” (P9). Seven of ten caregivers brought up school as a driver of QoL without prompting with one stating that QoL could be captured with questions like “His daily activity? Does he go to school? How, how does he do in school? Is it through virtual or is it actually regular schooling?” (P2). No caregivers brought up the domains of annoyance or sleep without prompting.

After going through the list of domains, eight of the caregivers were asked to prioritize which were the most important. Seven of eight caregivers prioritized happiness as the key determinant of QoL. One caregiver cited the combination of “happiness, activity, and sleep” (P4). The caregiver who did not cite happiness as most important mentioned a combination of activities, daily routine, and school as the most important determinants of QoL. This combination seemed to imply sense of similarity to other children without intestinal failure with that parent stating that the most important thing in assessing QoL is “just [her child] being able to participate and just basic things. And that includes, you know, school, hopefully, one day, eating normally, like, just foods that everybody eats, which right now is not an option. But just, like, moving towards those things that he feels — I don’t wanna say more normal cuz I feel like that's the wrong way to say it — but that he can do things, I guess, that his sister can do” (P3).

## Discussion

In this study, we interviewed parents of children with intestinal failure to determine whether their preferences around quality-of-life. We found that most parents felt that a combination of sadness, daily routine, and activities captures the essence of QoL with sadness alone being the most important single factor. However, once prompted with the nine domains of the CHU9D, we found that most parents agree with those nine domains with only slight disagreement with the domains of tiredness and annoyance. With tiredness, much of the question may be whether this represents a distinct construct from sleep. With annoyance, it was felt that this could not be reliably distinguished from normal behavior, especially during adolescence.

These results will play a critical role in shaping how we approach health utility measurement in children with intestinal failure moving forward. Several pediatric studies have examined the cost-utility of interventions such as teduglutide or transplant in pediatric intestinal failure [4], [5], [6], [7]. These studies have been limited by using the published data on health utilities in adults and attempting to adapt these to pediatrics [9]. However, we cannot rely on adult data to study effects on children. We have already seen no differences in QoL between children on and off parenteral nutrition, which is a stark contrast to the data from adults [2]. By identifying a tool to measure health utility in children with intestinal failure, we may now begin to redefine health utility values in pediatric intestinal failure to improve the accuracy of these cost-utility studies and their relevance for decision-making.

The finding that nearly all parents who were asked stated that sadness was the single most important domain presents another unique opportunity. Currently, the CHU9D is validated such that all nine domains are required to map onto a utility value set. Perhaps, sadness alone could provide sufficient discrimination regarding overall QoL. If not, there is potential for a short form that addresses sadness, school, daily routine, and activities, as these were identified by most parents without prompting. Exploring how to simplify data collection for health utilities may be crucial for successful study, especially in a rare disease population with a limited potential sample size.

As important as determining the most important factors, it seems that annoyance may be the least important factor for these caregivers. As parents suggested, older children may display annoyance even if that does not reflect a decrement to QoL. Both sleep and annoyance were not brought up by a single parent without prompting. These domains may not distinguish between QoL levels.

The CHU9D has been validated in other chronic gastrointestinal disorders such as pediatric inflammatory bowel disease (IBD) [18]. However, although the IBD and intestinal failure populations share some similarities, there are unique characteristics of patients with intestinal failure that can directly affect their QoL, such as the need for central venous catheters for administration of parenteral nutrition, the presence of feeding tubes, and enterocutaneous stomas. The impact of these medical devices on QoL may or may not be captured by the CHU9D. Therefore, further research is needed to assess the validity, reliability, and responsiveness of the CHU9D in detecting long-term change in preference-based health-related QoL in children with intestinal failure.

Another challenge specific to pediatric intestinal failure is that most patients are diagnosed in the first year of life [19], while the CHU9D was developed and validated for children aged 7–17 years old [12]. Preference based measures of QoL attempt to capture QoL data from a wide range of ages at various development stages. However, there are cognitive and practical limitations for obtaining QoL measures in younger children. This leads to a frequent scenario in which adult caregivers may be responding for their children based on their own preferences, and at time biases, about what living with disability may feel like. The role of ableism in affecting these responses requires further exploration. Furthermore, QoL dimensions should be age-appropriate for the respondent’s developmental stage to avoid ceiling and floor effects [20]. As such, additional validation may require purposively sampling caregivers with children at various ages and stages in their intestinal rehabilitation journey.

While the primary objective of this study was to evaluate the CHU9D, it is important to consider other health utility measurement tools as potential alternatives. The domains of sadness, daily routine, and activities are well-covered in the EuroQol-5D-Youth (EQ-5D-Y) [21], [22], [23], [24]. However, school is not included as a separate domain. The 16D health utility tool has sixteen domains, which does not seem to be consistent with the preferences of parents [25]. Thus, it seems both the EQ-5D-Y and CHU9D may be reasonable options for health utility assessment. Given the importance of school mentioned by most parents, we currently prefer the CHU9D.

Despite an adequately sized qualitative study, this work showcases only the viewpoints of a select group of parents of children with intestinal failure. These parents were available and motivated enough to participate, which may reflect a selection bias towards those with better QoL at this time. These results are thought to be reflective of the intestinal failure community but inclusion of the preferences of parents from other centers and from those not followed by an intestinal rehabilitation program may produce additional insights. Furthermore, the study only included parents and did not directly obtain the input of children and adolescents with intestinal failure. It is likely that their opinions may differ from the parents, especially as studies examining QoL have shown differences between self-reported and parent-proxy measures of health status in children [26], [27]. In particular, children may have a very different view of the importance of feeling annoyed or of the importance of school in determining QoL. Future work will include direct interviews with children and adolescents to determine if they corroborate our findings regarding the appropriateness of the CHU9D.

## Conclusion

In summary, parents of children with intestinal failure expressed similar preferences to the domains to assess QoL as what can be found in the CHU9D. They particularly stress the importance of sadness as well as daily functioning through school, activities, and daily routine. We recommend that the CHU9D be used to measure health utilities in pediatric intestinal failure.

## Ethical clearance

Ethics approval has been obtained by the University of Pittsburgh Institutional Review Board (STUDY21100136).

## Patient's/ Guardian's consent

Verbal consent obtained from all participants.

## Funding

Dr. Raghu was supported by the NASPGHAN Foundation/Alcresta Award for the Study of Pediatric Pancreatic Disease and Malabsorption and the National Center for Advancing Translational Sciences of the 10.13039/100000002National Institutes of Health under Award Number KL2TR001856. The content is solely the responsibility of the authors and does not necessarily represent the official views of the National Institutes of Health.

## CRediT authorship contribution statement

**Lisa Lakkis:** Writing – review & editing, Project administration, Formal analysis. **Flor de Abril Cameron:** Writing – review & editing, Methodology, Investigation, Data curation. **Daniela Gattini Valdes:** Writing – review & editing, Methodology, Conceptualization. **Beverly Kosmach-Park:** Writing – review & editing, Supervision, Investigation, Conceptualization. **Janel Hanmer:** Writing – review & editing, Supervision, Methodology, Conceptualization. **Vikram Raghu:** Writing – review & editing, Writing – original draft, Visualization, Validation, Supervision, Software, Resources, Project administration, Methodology, Investigation, Funding acquisition, Formal analysis, Data curation, Conceptualization.

## Declaration of Competing Interest

The authors declare the following financial interests/personal relationships which may be considered as potential competing interests: Vikram Raghu reports financial support was provided by Alcresta Therapeutics Inc. Vikram Raghu reports a relationship with Takeda Pharmaceuticals USA Inc that includes: consulting or advisory. If there are other authors, they declare that they have no known competing financial interests or personal relationships that could have appeared to influence the work reported in this paper.
